# Analysis of Tumor Heterogeneity and Cancer Gene Networks Using Deep Sequencing of MMTV-Induced Mouse Mammary Tumors

**DOI:** 10.1371/journal.pone.0062113

**Published:** 2013-05-14

**Authors:** Christiaan Klijn, Marco J. Koudijs, Jaap Kool, Jelle ten Hoeve, Mandy Boer, Joost de Moes, Waseem Akhtar, Martine van Miltenburg, Annabel Vendel-Zwaagstra, Marcel J. T. Reinders, David J. Adams, Maarten van Lohuizen, John Hilkens, Lodewyk F. A. Wessels, Jos Jonkers

**Affiliations:** 1 Division of Molecular Pathology, The Netherlands Cancer Institute, Amsterdam, The Netherlands; 2 Division of Molecular Genetics, The Netherlands Cancer Institute, Amsterdam, The Netherlands; 3 Division of Molecular Carcinogenesis, The Netherlands Cancer Institute, Amsterdam, The Netherlands; 4 Delft Bioinformatics Lab, Delft University of Technology, Delft, The Netherlands; 5 Wellcome Trust Sanger Institute, Hinxton, Cambridge, United Kingdom; Sanford Burnham Medical Research Institute, United States of America

## Abstract

Cancer develops through a multistep process in which normal cells progress to malignant tumors via the evolution of their genomes as a result of the acquisition of mutations in cancer driver genes. The number, identity and mode of action of cancer driver genes, and how they contribute to tumor evolution is largely unknown. This study deployed the Mouse Mammary Tumor Virus (MMTV) as an insertional mutagen to find both the driver genes and the networks in which they function. Using deep insertion site sequencing we identified around 31000 retroviral integration sites in 604 MMTV-induced mammary tumors from mice with mammary gland-specific deletion of *Trp53*, *Pten* heterozygous knockout mice, or wildtype strains. We identified 18 known common integration sites (CISs) and 12 previously unknown CISs marking new candidate cancer genes. Members of the *Wnt*, *Fgf*, *Fgfr*, *Rspo* and *Pdgfr* gene families were commonly mutated in a mutually exclusive fashion. The sequence data we generated yielded also information on the clonality of insertions in individual tumors, allowing us to develop a data-driven model of MMTV-induced tumor development. Insertional mutations near *Wnt* and *Fgf* genes mark the earliest “initiating” events in MMTV induced tumorigenesis, whereas *Fgfr* genes are targeted later during tumor progression. Our data shows that insertional mutagenesis can be used to discover the mutational networks, the timing of mutations, and the genes that initiate and drive tumor evolution.

## Introduction

With the advent of next-generation DNA sequencing technologies the mutational landscape of several tumor types has been defined revealing that there are numerous genetic paths to malignancy [1]. Tumor heterogeneity further contributes to this complexity [2], [3], thus complicating our ability to distinguish driver mutations from passengers. Mouse tumor models present a clean, reproducible *in vivo* system to study the contribution of driver genes to tumorigenesis and to define their underlying biological mechanisms of action [4].

Insertional mutagenesis (IM) employing retroviruses or transposons has been one of the main tools for inducing tumors in mice [5]–[8]. Mouse Mammary Tumor Virus (MMTV) is a slow-transforming retrovirus that has been used to study mammary tumorigenesis in mice. This virus causes mammary tumors by integration of its proviral DNA in or near cancer genes. Repeated cycles of insertional mutation and clonal expansion leads to mammary tumors carrying multiple clonal and sub-clonal MMTV integrations, including mutations linked to both driver genes as well as passenger mutations.

Molecular cloning of the proviral insertions in MMTV-induced mammary tumors led to the discovery of the first MMTV Common Insertion Site (CIS) and the associated gene *Wnt1* (originally called *Int1*) in 1982 [9]. It was found that MMTV insertions near the *Wnt1* gene promoted mammary tumor development via activation of Wnt1, the founding member of the Wnt signaling pathway. Soon after the discovery of *Wnt1* another oncogene, *Fgf3* (originally termed *Int2*) was identified. Further research showed that this member of the fibroblast growth factor gene family effectively collaborates with *Wnt1* in tumor formation [10]. Following the promise of these early studies, the increasing popularity of MMTV as a screening system resulted in the discovery of several additional genes implicated in cancer development [11]–[17].

The heterogeneity and progression of MMTV-induced tumors can be assessed by analyzing the relative abundance (“clonality”) of individual insertions in a given sample. Highly abundant insertions indicate early, initiating insertion events and lowly abundant insertions indicate events that occur later during tumor development, analogous to recent studies of single nucleotide mutations in human tumors [2], [3]. Previously used PCR-based approaches are unable to reliably quantify the clonality of insertion sites due to sequence amplification biases. We therefore developed a method for simultaneous identification and quantitative assessment of clonality of insertional mutations called Shear-Splink [18]. We applied this method to analyze a large set of MMTV-induced tumors from two wild-type mouse strains (BALB/c and FVB/N) and two genetically engineered mouse (GEM) models of breast cancer: the *Pten^+/−^* strain [19] and the *K14Cre;Trp53* model [20]. We used the resulting dataset to address four key questions: Firstly, can we identify novel MMTV CISs and thereby extend the repertoire of candidate cancer genes associated with these models? Secondly, can we identify genotype specific driver genes in each of the genetic backgrounds? Thirdly, can we identify co-occurring or mutually exclusive relationships between CISs and thus define functional relationships between the associated driver genes? Finally, can we generate a tumor progression model from the clonality information derived from the sequence reads of the individual insertions? Such a model would specify the order of events based on the insertion profile and shed light on functional relationships between genes involved in MMTV-induced mammary tumorigenesis.

## Materials and Methods

### Mouse models used for MMTV infection

Newborn BALB/c/He/A (denoted BALB/c) mice were infected with MMTV by foster nursing on C3H/A females harboring the milk transmitted MMTV [21]. Infected BALB/c female mice develop mammary tumors with high incidence (>95% before the age of 1 year). The virus-infected animals were denoted BALB/c^+^ mice. In this study we used two transgenic mouse lines and their wild-type controls: a strain conditionally deficient for *Trp53* in mammary epithelial tissue on a BALB/c background (Balb/c *K14Cre;Trp53^F/F^*) and a germ-line heterozygous knockout for *Pten* on an FVB/N background.

The BALB/c *K14Cre;Trp53^F/F^* mouse strain was generated by nine consecutive backcrosses of *K14Cre;Trp53^F/F^* animals on a mixed 129P2/Ola and FVB/N background [22] to BALB/c mice. The resulting Balb/c *K14Cre;Trp53^F/F^* strain showed only a low mammary tumor incidence before the age of 300 days. These conditional *Trp53* knockout mice were infected by MMTV through foster nursing by BALB/c^+^ females.

For comparison of MMTV induced tumorigenesis between wild type and *Pten* ^+/−^ mice, the conditional *Pten* knockout allele [23] was used to generate *Pten* ^+/−^ FVB/N mice. *Pten* ^+/−^ mice were crossed with FVB/N wild-type mice. The female littermates in the progeny, both wild-type and heterozygous for *Pten*, were infected with MMTV by foster nursing on BALB/c^+^ females harboring the milk transmitted MMTV.

All MMTV-infected animals were monitored for the development of mammary tumors which were isolated when approximately 1 cm in diameter. This study was carried out in strict accordance with the Dutch Code of Practice for Research with Laboratory Animal in Cancer Research. The protocol was approved by the local experimental Animal ethics Committee (DEC) (Permit Numbers: 04065, 08061, 03008) and all efforts were made to minimize suffering. All mice were sacrificed when the tumor reached 1 cm^3^ (end point) by carbon dioxide. Kaplan-Meier plots of mouse life span were plotted and log-rank tests were calculated using the survival package as implemented in the statistical programming language R.

Cre mediated deletion of the floxed *Trp53* alleles in tumors from *K14Cre;Trp53^F/F^* mice was assessed by Southern blot analysis as described previously 2001 [24]. Briefly, high molecular weight genomic DNA from MMTV induced tumors was digested with BglII. DNA fragments were separated on 0.6% agarose gels and blotted on to nitrocellulose filters. A PCR labeled 700 nt XbaI fragment corresponding to exon 11 of *Trp53* was used as probe to detect deleted and non-deleted alleles. Cre mediated deletion of exon 2–10 of the floxed *Trp53* allele could be assessed based on a mobility shift of the *Bgl*II DNA fragment. Approximately 50% of the tumors showed bi-allelic loss of the floxed exons. Only those tumors were used for IM analysis.

### Shear-Splink method for insertion site mapping

To sequence the insertion sites in tumors we used the Shear-Splink protocol as previously described [18]. Briefly, DNA was sheared to 100–1000 bp fragments, which were blunt-ended and ligated to splinkerette adapters. A primary PCR was performed to specifically amplify viral-to-host junction fragments. A second PCR was performed to introduce the barcode sequences and the adapters needed for 454 sequencing. These PCR products were pooled and sequenced by 454 sequencing. The raw sequencing data has been deposited at the Sequence Read Archive under accession number ERP002483.

### Data analysis

Data analysis methods are detailed in the Supporting Methods (Text S1).

### RNA sequencing

RNA was extracted from 4 tumors carrying *Hbegf* insertions, and 4 cases carrying a *Myb* insertion. We determined FPKMs for both *Hbegf* and *Myb* using Cufflinks [25] after alignment using BWA [26]

## Results

### MMTV insertional mutagenesis in wild-type and Trp53 or Pten mutant mice

**Figure 1:**
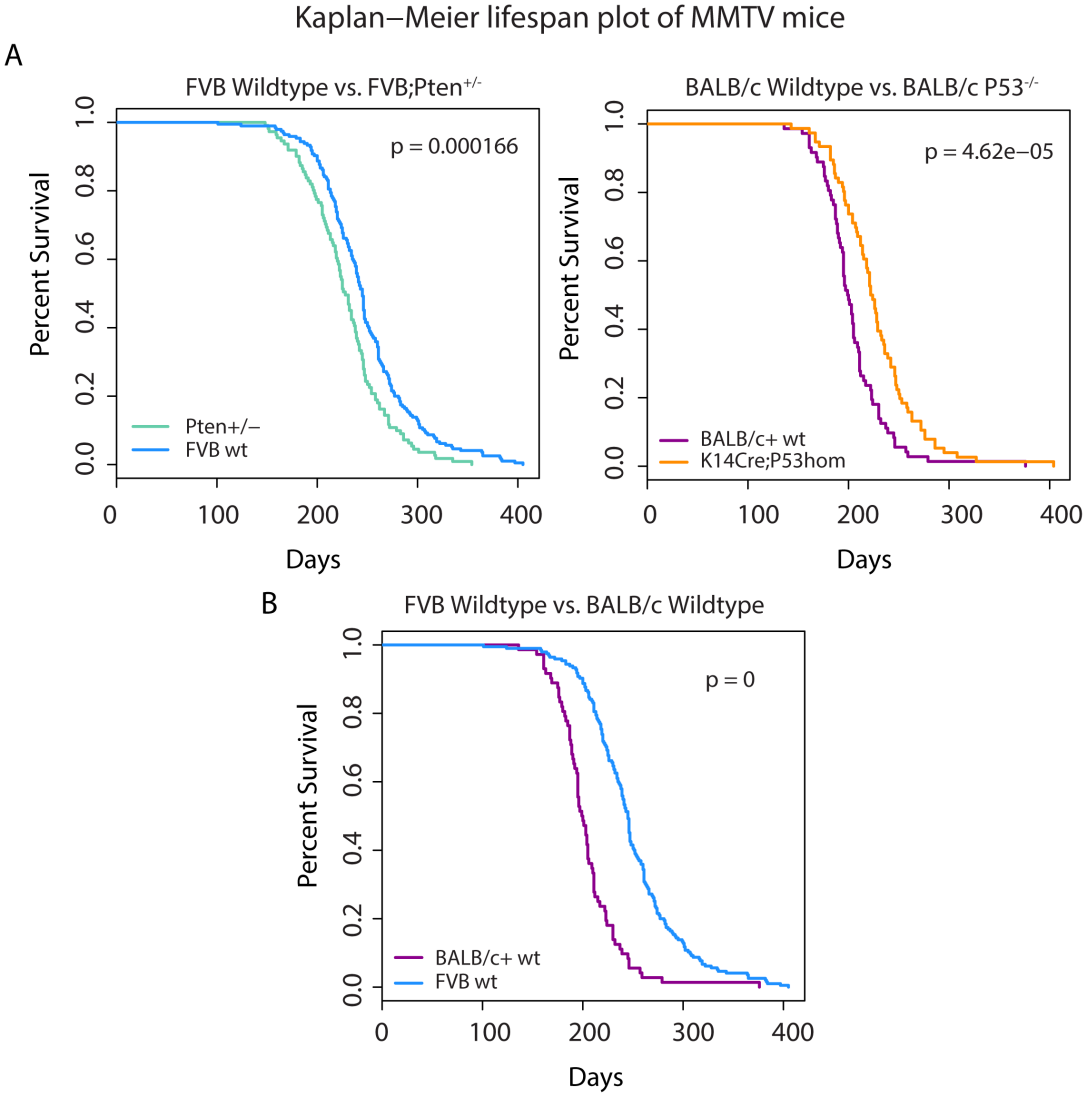
Kaplan Meier curves for the four strains of mice used in this study. Each graph represents a comparison between two cohorts. A pairwise log-rank test was performed for all graphs to determine whether there are significant lifespan differences between the cohorts plotted in each graph. P-values are shown in the upper right corner. **A.** Within each specific mouse background strain (FVB or BALB/c+) we compared the MMTV-infected wild-type cohort with the infected genetically engineered line (either *Pten* heterozygous for the FVB cohort or *Trp53* deficient for the BALB/c+ cohort). **B.** The difference in lifespan between the two wild-type strains is shown here.

In order to assess tumor heterogeneity and identify cancer gene networks in MMTV-induced mouse mammary tumors, we performed a high-throughput, large-scale Insertional Mutagenesis study in combination with deep sequencing in a large cohort of mice from 4 different genetic backgrounds. A schematic overview of our approach is depicted in Figure S1. We analyzed cohorts of mice of two different genetic backgrounds: FVB/N (hereafter referred to as FVB) and BALB/c. For each genetic background we acquired tumors from both the wild-type strain as well as from specific genetically engineered mouse (GEM) models. Within the FVB background, mammary tumors were harvested from MMTV-infected wild-type mice and *Pten^+/−^* heterozygous knockout mice. Within the BALB/c background MMTV-induced tumors were obtained from wild-type animals and *K14cre;Trp53^F/F^* mice with epithelium-specific deletion of p53. As can be seen in Figure 1a, there is a lifespan difference between the wild-type controls and the matched GEM models, indicating an interaction between MMTV-induced tumorigenesis and the genetically engineered mutation. Interestingly, the median latency of MMTV-induced tumor development was decreased in the *Pten^+/−^* cohort, but increased in the *K14cre;Trp53^F/F^* cohort when compared to their wild-type controls. This observation led us to hypothesize that the MMTV insertions might hit genes that collaborate with *Pten* haploinsufficiency in *Pten^+/−^* mice. In contrast, MMTV infection might negatively influence malignant transformation of *Trp53^−/−^* mammary epithelial cells or vice versa. There is also a large difference in lifespan between MMTV-infected wild-type FVB/N and BALB/c mice (Figure 1b). This could simply indicate that the virus has slower replication in the FVB strain compared to the BALB/c strain, or it could indicate the presence of BALB/c alleles that promote MMTV-induced tumorigenesis. In support of the latter, BALB/c mice contain a hypomorphic allele of the *Cdk2na* tumor suppressor gene [27].

To map the MMTV insertion sites we used our Shear-Splink protocol [18] to extract and amplify virus-host DNA junction fragments containing the MMTV 5′ LTR as well as the adjacent mouse genomic sequence. We barcoded the fragments allowing us to pool up to 48 individual tumors and sequence them on the 454 Genome Sequencer FLX system. Raw sequences were preprocessed using custom Perl scripts. We identified the genomic sequence from the reads and mapped them to the C57BL/6J (MGSC37) reference genome. For each read we confirmed the presence of the 5′ end of viral sequence as well as the presence of the DNA barcode to de-convolve the pools into individual tumor data. One of the unique aspects of our approach is that we are able to quantify the clonality of insertions by counting the number of unique tumor-host DNA junction fragments marked by unique ligation points (LPs) between the mouse genomic sequence and the splinkerette adapter. Since the number of unique LPs corresponds to the number of cells carrying the corresponding MMTV insertion, the LP count can be used as a measure for the relative clonality of individual MMTV insertions within each tumor [18].

Several additional filtering steps were performed as described in the Methods section and depicted in Figure S1 before the data was entered into the Insertional Mutagenesis Database (iMDB; http://imdb.nki.nl). After filtering we were left with 30942 integrations in 604 tumors. Any insertions that have *n* unique LPs are guaranteed to have been present in at least *n* independent cells. To enrich for insertions that had integrated in more than one tumor cell, we disregarded insertions with only one LP. Using this filter we reduced the data to 6605 unique insertions in 600 tumors (4 tumors carried only insertions with 1 LP). This is a reduction of 78% in the total number of insertion sites, but it only constitutes a reduction of 21% in the total number of reads, suggesting that filtering against insertions with single LPs effectively reduces the number of background mutations in our data.

### CIS analysis of MMTV integration sites

**Figure 2:**
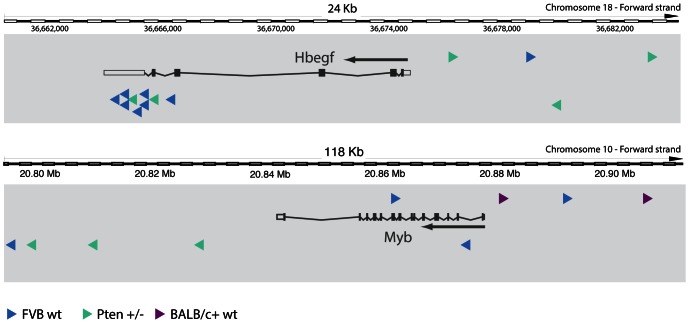
MMTV integrations in two novel CISs. The putative target gene is shown, with the arrow indicating the transcriptional direction. Arrowheads indicate the genomic location of the viral integrations, with the direction of the arrow indicating the viral transcription direction. Colors indicate the cohort from which the integrations were recovered.

We determined global CISs for all tumors in the dataset. To determine significant CISs we used the Gaussian Kernel Convolution framework [28] implemented in the iMDB. Most of the tumors from wild-type mice contributed at least one insertion to a CIS (Table 1). This percentage is lower for the predisposed backgrounds. This is most probably due to the fact that some of the *K14Cre;Trp53^F/F^* and *Pten^+/−^* mice develop mammary tumors that are not driven by MMTV insertional mutagenesis. In total, we found 30 significant CISs, of which 18 were already known and 12 were novel (Table 2). All insertions associated with a CIS are accessible via the iMDB. We manually assigned potential target genes to these CISs. As an illustration we show the two most frequent novel CISs and the mapping of the MMTV insertions in these CISs with respect to the target gene in Figure 2. Both *Hbegf* and *Myb* are very plausible candidate target genes as the MMTV integrations are very likely to enhance expression (upstream and downstream integrations) or stabilize the mRNA by premature transcription termination and concomitant removal of mRNA destabilizing motifs due to integrations in the 3′ UTR (in the case of *Hbegf*). Moreover, both *Hbegf* [29], [30] and *Myb* [31] have been previously implicated in cancer. Finally, the expression of both Hbegf and Myb is high in samples carrying the integration, showing that these genes are direct targets of the viral integration (Figure S2).

**Table 1:** Mouse tumors contributing an insertion to a common insertion site.

Genotype	Strain Background	Number of tumors with insertions in a CIS^1^ (n (% of total))
Wt	BALB/c	78 (98%)
*K14Cre;Trp53^f/f^*	BALB/c	46 (78%)
Wt	FVB	246 (93%)
*Pten^+/−^*	FVB	166 (83%)

This table gives an overview of the different genotype/strain combinations and the number of tumors that contain at least one.

1 Common Insertion Site (CIS).

**Table 2:** Significant known and novel common insertion sites.

Chromosome	Start (bp)	End (bp)	Curated Target Gene	Tumors with Insertion	CGC	Amplified in Cell Lines (CONAN)	Deleted in Cell Lines	COSMIC (% mutated of samples tested)
Known CISs								
**chr15**	98520001	98712001	*Wnt1*	270	N	3	0	0
**chr7**	151923001	152121001	*Fgf3*	195	N	28	0	0
**chr19**	45606001	45867001	*Fgf8*	135	N	0	0	0
**chr11**	59004001	59175001	*Wnt3a*	84	N	5	0	1%
**chr11**	103557001	103779001	*Wnt3*	59	N	1	0	0
**chr7**	137232001	137505001	*Fgfr2*	51	Y	5	0	3%
**chr15**	42954001	43134001	*Rspo2*	48	N	6	0	2%
**chr18**	4275001	4395001	*Map3k8*	30	N	0	0	0
**chrX**	138084001	138234001	*Irs4*	23	N	1	1	1%
**chr10**	29211001	29361001	*Rspo3*	25	N	0	0	NA
**chr7**	149778001	149895001	*Igf2*	19	N	0	0	0
**chr2**	10242001	10353001	*Sfmbt2*	17	N	0	0	0
**chr5**	75501001	75594001	*Pdgfra*	14	Y	5	0	7%
**chrX**	7464001	7554001	*Eras*	13	N	0	0	1%
**chr18**	61179001	61236001	*Pdgfrb*	7	Y	1	0	1%
**chr10**	29490001	29529001	*Rspo3 enhancer*	6	N	0	0	NA
**chr6**	127188001	127203001	*Fgf6/Fgf23*	4	N	3 - Fgf6/4 - Fgf23	0	0.5% - Fgf6/1% Fgf23
**chr13**	119487001	119520001	*Fgf10*	5	N	14	0	1%
New CISs								
**chr8**	26397001	26667001	*Fgfr1* 1	30	Y	5	0	1%
**chr18**	36621001	36714001	*Hbegf*	15	N	0	0	0
**chr10**	20820001	20898001	*Myb* 2	9	Y	5	0	0
**chr11**	121536001	121599001	*Metrnl/Ptchd3*	8	N	2	0	0% - Metrnl/Ptchd3
**chr15**	74901001	74943001	*Reg. Feat*	6	N	NA	NA	NA
**chr6**	103575001	103623001	*Chl1*	6	N	0	0	1%
**chr4**	124671001	124716001	*Rspo1*	6	N	3	0	0
**chr3**	97782001	97821001	*Notch2*	6	Y	2	0	2%
**chr5**	34044001	34068001	*Fgfr3*	5	Y	2	0	26%
**chr11**	3051001	3072001	*Sfi1*	5	N	0	0	1%
**chr14**	68478001	68511001	*Dock5*	5	N	0	2	4%
**chr6**	23181001	23217001	*Fezf1*	5	N	10	0	3%

This table gives an overview of the significant CISs and their potential target genes.

1 Although Fgfr1 has not been found as a common insertion site in [11], the authors do mention finding one insertion near the gene.

2 The *Myb* CIS is a merge of two overlapping CISs (upstream and downstream of the *Myb* gene).

Many of the CISs we recovered are canonical CISs for MMTV insertional mutagenesis. In MMTV-induced mammary tumors, proviral insertions are often found near *Wnt* gene family members (*Wnt1*, *Wnt3* and *Wnt3a*), growth factor related genes (*Fgf* and *Fgfr* genes, *Pdgfr* genes and *Igf2*), R-spondin gene family members (*Rspo1*, *Rspo2* and *Rspo3*) and mitogen signaling pathway genes (*Eras* and *Map3k8*) [11]. We identified several novel CISs in these families that were not previously identified: *Hbegf*, *Rspo1* and *Fgfr3*. These novel targets can only be recovered in a large study since they occur much less frequently compared to the previously identified family members. We also recovered several rare CISs that are likely to be true MMTV hits because the candidate target genes belong to gene families which also include members that are frequent MMTV targets. This finding supports the validity of other rare, but significant novel CISs we identified in our screen, such as *Sfi1*, *Dock5* and *Fezf1*. Indeed, the human homolog of *Fezf1* (known as *ZNF312B*) has been described as an oncogene in gastric cancer [32]. Furthermore, several known and novel target genes were found to be recurrently amplified in a panel of over 700 human cancer cell lines (http://www.sanger.ac.uk/cgi-bin/genetics/CGP/conan/search.cgi) or listed as mutational targets in the COSMIC database (http://www.sanger.ac.uk/genetics/CGP/cosmic/). This shows that genes associated with CISs in MMTV-induced mammary tumors may also be relevant in human cancer.

### Genotype-specific CISs

Besides finding novel MMTV CISs we were interested in finding genotype-specific insertions. MMTV infected *Pten* ^+/−^ mice develop mammary tumors faster than their wild-type controls, suggesting that MMTV insertions might mutate cancer-relevant genes that collaborate with Pten haploinsufficiency in mammary tumorigenesis. If this is the case we would expect to find enrichment for specific CISs in MMTV-induced *Pten^+/−^* mammary tumors, compared to control tumors. However, we could not find any significant association with either background in our study (Table 2). This suggests that MMTV integrations occur in or near the same cancer driver genes, regardless of *Trp53* or *Pten* status. There was also a large difference in lifespan between the MMTV-infected FVB and BALB/c wild-type mice (Figure 1b). Also here, we could not find any significant association between CISs and strain background. Although we observed insertions near *Hbegf* only in the FVB background (Figure 2), this difference was not statistically significant, probably due to the low incidence of insertions near this gene. It does however hint towards the possibility that activation of *Hbegf* may be oncogenic in FVB mice but not BALB/c mice.

### Co-occurrence and mutual exclusivity between CISs

**Figure 3:**
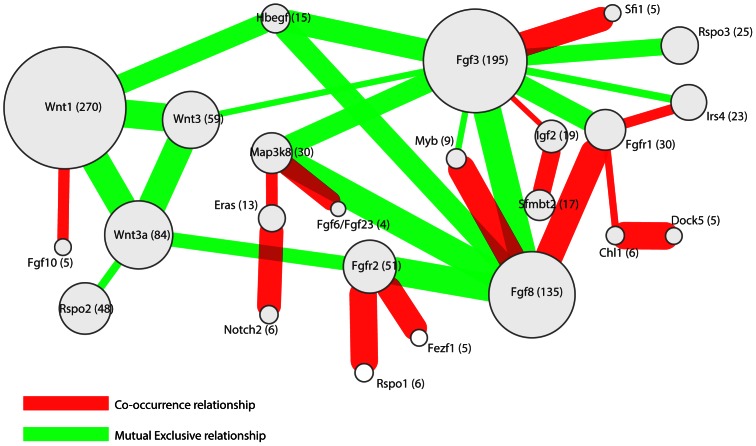
A network of significant co-occurring and mutually exclusive common insertion sites (CISs). CISs are indicated by their manually curated target gene. Red edges indicate a co-occurrence relationship, while green edges indicate a mutually exclusive relationship. The number in parenthesis and the size of the nodes indicate the number of tumors with a viral insertion in the relevant CIS. The thickness of the edges is a measure of the significance of the relationship between the nodes.

Our analysis of common insertion sites yielded several novel but rarely tagged loci. We analyzed the insertion patterns for all CISs across all tumors to establish relationships between the insertion patterns in these CISs. Insertions can exhibit a mutually exclusive integration pattern, which could signify functional redundancy between the target genes as has been shown for *Myc* and its paralog *N-myc* [33]. Conversely, co-occurring insertions may be observed in cases where the oncogenic effect of both insertions is synergistic. To identify such functional relationships, we determined co-occurrence and mutual exclusivity between the statistically significant CISs in our study as described in the Methods section (Figure 3).

Several observations can be made from this analysis. Firstly, significant co-occurrence occurs primarily between infrequent insertions and mutual exclusivity mainly between highly frequent insertions. This bias is most probably due to the way of testing, which is underpowered for low insertion frequencies. Infrequent, mutually exclusive insertions need a larger sample size to become significant, while infrequent, co-occurring insertions can quickly become significant.

Secondly, interesting relationships were found between the members of the *Fgf* ligand family and their receptors, the *Fgfr* genes. For example, the FGFR ligands FGF8 and FGF3 appear to have a reciprocal preference for the receptor FGFR1, since *Fgf3* insertions are mutually exclusive with *Fgfr1* insertions whereas *Fgf8* insertions significantly co-occur with *Fgfr1* insertions. This might indicate preferential binding partners for the different ligands and a selective advantage for up-regulation of both the ligand and its matched receptor.

**Figure 4:**
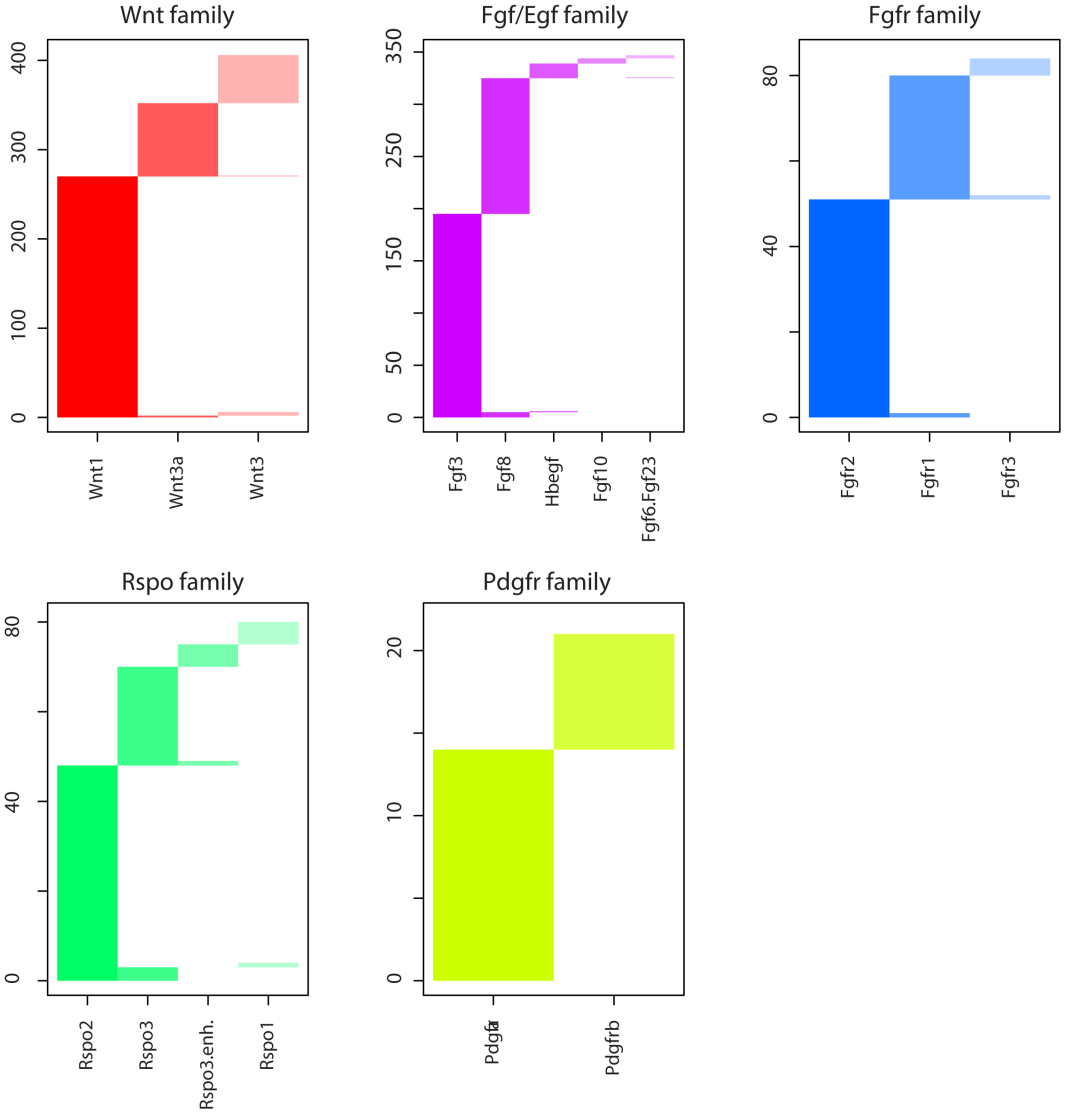
Insertion patterns of CIS gene families. For each of five families the columns indicate which tumors contained an insertion for that specific member of a family. Rows indicate specific tumors.

Finally, we observed mutual exclusivity between members of individual gene families (e.g. *Wnt* genes and *Fgf-Hbegf* genes). Plotting of cumulative insertion patterns for five distinct families of CIS genes (*Wnt*, *Fgf/Egf*, *Fgfr*, *Rspo* and *Pdgfr*) revealed a typical mutually exclusive integration pattern for genes within each family (Figure 4), showing that MMTV infected cells gain little to no selective advantage from MMTV insertions near multiple members of the same gene family. Based on these results we decided to look for relationships between CIS gene families instead of single CIS genes.

### Model for MMTV Tumor Progression

Our data show that MMTV preferentially targets a rather limited group of genes and gene families. If we restrict ourselves to CIS genes and CIS families that are affected by MMTV insertions in ten or more tumors, we are left with only 11 groups of CIS genes (Table 2). We were interested to see if we could find a difference in clonality between these gene groups. When comparing insertions near two members of these groups within one tumor, the one with more clonal insertions will also have a higher clonality score based on the unique LPs counts. This could indicate an earlier event and/or a more potent hit resulting in stronger positive selection. We tested for all 11 groups of CIS genes all pairs of insertions that occurred in the same tumor in order to test if members of one group have a consistently higher clonality scores than members of another group, when co-mutated in the same tumor (see Methods for details).

**Figure 5:**
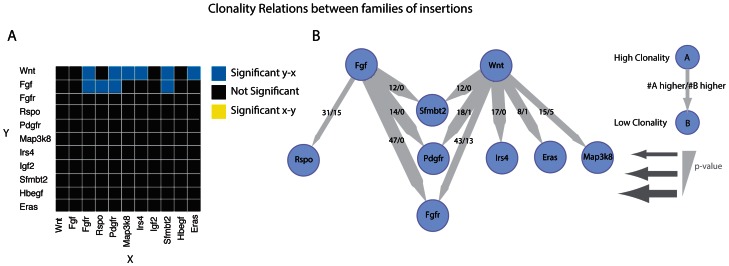
Analysis of clonality between different families of genes. **A.** A heatmap of all combinations of gene families and single genes not assigned to a family. Significant difference in clonality for each family are calculated using a binominal test for all samples that are co-inserted in that specific gene (family) pair. Blue squares indicate a significant clonal relation from the group indicated on the Y-axis to the group indicated on the X-axis. Yellow squares indicate a significant clonal relationship from the X-axis to the Y-axis. Black squares indicate no significant relation. **B.** A network view of the heatmap in A. showing only significant (P<0.05) clonality relationships. An edge points from the more clonal gene(family) to the lesser clonal gene(family). The thickness of an edge is a measure of the significance of the clonality relation. For the fraction displayed on the edges, the numerator represents the number of times the parent node had a higher clonality score while the denominator represents the number of times the child node had a higher clonality score, in a tumor that contained insertions in both nodes.

Figure 5a shows a heatmap with the CIS gene/family pairs that had a significant clonality relation according to the binominal test. This analysis reveals significant relationships between the *Wnt/Fgf* gene families (higher clonality) and *Rspo*, *Sfmbt2*, *Pdgfr*, *Fgfr*, *Irs4*, *Eras* and *Map3k8* (lower clonality). In Figure 5b we visualized only these significant relationships together with their directionality. From this data-driven model we can formulate a simple progression model for MMTV-induced mouse mammary tumorigenesis. Tumor-initiating MMTV integrations are most likely to occur near an *Fgf* or *Wnt* gene, whereas insertions near other CIS genes are secondary events. For all other relations tested there is either no clear clonality relation or there are no tumors in which they are co-mutated. In all 47 tumors that showed co-mutation of *Fgf* and its receptor *Fgfr*, the *Fgf* insertion was more clonal, showing that the FGF ligand is always activated earlier than the FGF receptor during MMTV tumor progression.

## Discussion

Recent studies have delved into the heterogenic make-up of human tumors [2], [3] showing that a steady accumulation of background mutations covers the fact that only a handful of driving mutations causes oncogenic differentiation. Using the mouse system, we were able to clearly define the driver genes for MMTV-induced mammary tumors and the order in which they get deregulated.

### MMTV insertional mutagenesis

In this study we analyzed a large cohort of mice that developed tumors through MMTV insertional mutagenesis. Within a dataset of 6600 non-background MMTV insertions from 600 tumors, we identified 30 common insertion sites encompassing 1271 of the 6600 insertions (19.3%). We used this dataset to address several questions regarding MMTV-induced mouse mammary tumorigenesis. Firstly, we wanted to see if we could identify novel MMTV CISs in this large dataset using our high-throughput Shear-Splink approach. We found that the 30 MMTV CISs target a limited number of well-defined gene families and very few other genes. In total, 53 MMTV CISs have been previously identified (Table S1). The overlap between this list of known MMTV CISs and our list is only 18 CISs. This means that 66% of the previously found CISs could not be confirmed in this study. Although it is possible that CISs with very subclonal insertions are not detected by our Shear-Splink method, we believe that our study is both less biased (through the use of DNA shearing instead of restriction enzyme cleavage) and more robust (since we measure many reads of the same insertion) than older studies based on isolation and Sanger sequencing of individual MMTV insertion sites. Also, the use of the LP score to filter background insertions from the data is a powerful method to limit false positive findings. While it is possible that our method generates false-negatives, it is perhaps more likely that some of the previously identified CISs are passengers or random integration sites. Our data suggest that MMTV-induced mammary tumorigenesis is a very specific disease involving a limited number of cellular target genes that may promote proliferation, survival and/or self-renewal of MMTV-infected mammary epithelial cells. As such, MMTV is not a very flexible insertional mutagenesis system and therefore probably not the best approach to identify novel candidate breast cancer genes in wild-type mice or tumor-predisposed GEM models. This notion is supported by the fact that we cannot find specific MMTV insertions in mammary tumor-prone mice with heterozygous *Pten* deletion or tissue-specific loss of *Trp53*, even though MMTV-infected *Pten* heterozygous mice developed mammary tumors faster than their wild-type counterparts. MMTV-induced tumorigenesis apparently profits from haploinsufficiency for *Pten* but mutation of specific collaborators is not required for this condition. Our results do not rule out that MMTV might show a different insertion pattern in mice with mammary gland-specific over-expression of a strong oncogene.

Despite the strong bias of MMTV towards activation of Wnt/Fgf family members, we could still identify several novel CISs for MMTV due to the large number of samples we included in our analysis. Some of the new CISs fall within the established target gene families, showing that our method is able to identify true positives even if they only occur in <1% of the samples, as is the case with *Fgfr3*. It can be expected that additional rare MMTV CISs can be identified by further increasing the sample-size of the study. However, with the diminishing returns and the increasing costs, this strategy is probably not preferable, especially in view of the rapidly decreasing costs of genomic sequencing of spontaneous tumors and the advent of novel transposon systems. Novel CISs that do not map near members of the canonical MMTV gene families, such as *Myb* and *Fezf1*, have been previously associated with cancer. Although *Myb* is a known common target for Mouse Leukemia virus [34] and implicated in human lymphoma [31], it has only recently been associated with breast cancer [31], [35]. Finding *Myb* as a common target in mouse mammary tumorigenesis adds to the evidence that *Myb* is a bona fide oncogene in breast cancer.

Our analysis of a large group of MMTV-induced mouse mammary tumors also allowed us to investigate relations of either mutual exclusivity or co-occurrence between the different MMTV CISs. We showed that within the five commonly targeted gene families (*Wnt*, *Fgf*, *Fgfr*, *Rspo* and *Pdgfr*) individual members showed a strong mutually exclusive mutation pattern, indicating that an MMTV insertion near one family member abolishes selective pressure for insertions near additional members of the same family. Although overexpressed *Fgf* and *Wnt* genes are synergistic in inducing mammary tumors in bitransgenic mice [36], we did not observe a significant co-occurrence of MMTV insertions in *Wnt* and *Fgf* family members. While we recovered many tumors with insertions near members of both gene families, we recovered almost equal numbers of tumors with an insertion near members of only one of the two families. These results suggest that combined activation of *Fgf* and *Wnt* might not be required for MMTV-induced tumorigenesis or that activation of *Fgf* and *Wnt* in MMTV-induced mammary tumors might also occur via mechanisms other than insertional mutations (i.e. point mutations, genomic rearrangements, copy number aberrations and/or epigenetic changes). In support of the latter notion, we could detect increased mRNA expression of *Fgf* and *Wnt* family members in several tumors with no detectable MMTV insertions near these genes (data not shown).

The strong mutual exclusivity among members of individual CIS gene families allowed us to group the targeted genes into families and interrogate relationships between insertions in the different CIS groups. We used the LP score that followed from our Shear-Splink analysis to calculate relative clonality in order to determine for all pairs of co-occurring insertions which of the two insertions was present in a higher number of tumor cells. The more clonal insertions have probably occurred earlier during tumor progression. Although the clonality score could be influenced by DNA copy number and the presence of multiple independent tumors in one location, we assume that in most cases the clonality score can effectively distinguish between early and late events. Our pair-wise analysis of the clonality scores showed that insertions near *Wnt* or *Fgf* genes are early events compared to insertional mutations near other gene families. Taken together, our observations indicate that MMTV induced mammary tumorigenesis is a very specific disease involving activation of a limited number of cellular target genes.

## Conclusions

This study is the largest MMTV insertional mutagenesis screen performed to date. Our high-throughput Shear-Splink method has shown that the pattern of recurrent insertions in MMTV-induced mouse mammary tumors is very specific and dominant over genetic background and tumor-predisposing mutations. Only a handful of gene families are targeted in a specific tumor progression program. What causes the specificity of MMTV towards these genes is unclear but it most likely involves complex interactions between MMTV and the host cell. Target gene specificity seems unlikely to be due to potential integration biases of the MMTV provirus, since the MMTV insertions near the major targets are spread over multiple kilobases and since different family members are targeted in a mutually exclusive fashion. After almost a century of MMTV research [21], MMTV-induced tumorigenesis appears to be a highly defined and potent genetic program.

## Supporting Information

Figure S1 **Schematic overview of the study.** Boxes depict application of protocols and arrows indicate the flow of the resulting products. The gray box indicates that all processes take place inside the insertional Mutagenesis Database.
(TIF)

Figure S2 **Expression of Myb and Hbegf in tumors with viral insertions near to these genes.** The RPKM gene expression values of the genes Myb and Hbegf have been plotted for 8 mammary tumors. Four of these tumors contained a MMTV insertion near Myb (shown with triangles) and four of the tumors contained a MMTV insertion near Hbegf (shown with circles). The color of the symbols represents the clonality of those insertions.
(TIF)

Table S1 **List of previously identified MMTV Common Insertion Sites.** This table lists previously identified MMTV CISs in the literature that were compared to the ones identified in this study.
(XLS)

Text S1 **Supplemental Methods.** This document describes the data analysis steps used in this study in more detail.
(DOC)
