# Supplementary figures and images for: Analysis of Tumor Heterogeneity and Cancer Gene Networks Using Deep Sequencing of MMTV-Induced Mouse Mammary Tumors
Source: PLoS One. 2013 May 14;8(5):e62113. doi: 10.1371/journal.pone.0062113 (PMC3653918; doi:10.1371/journal.pone.0062113)

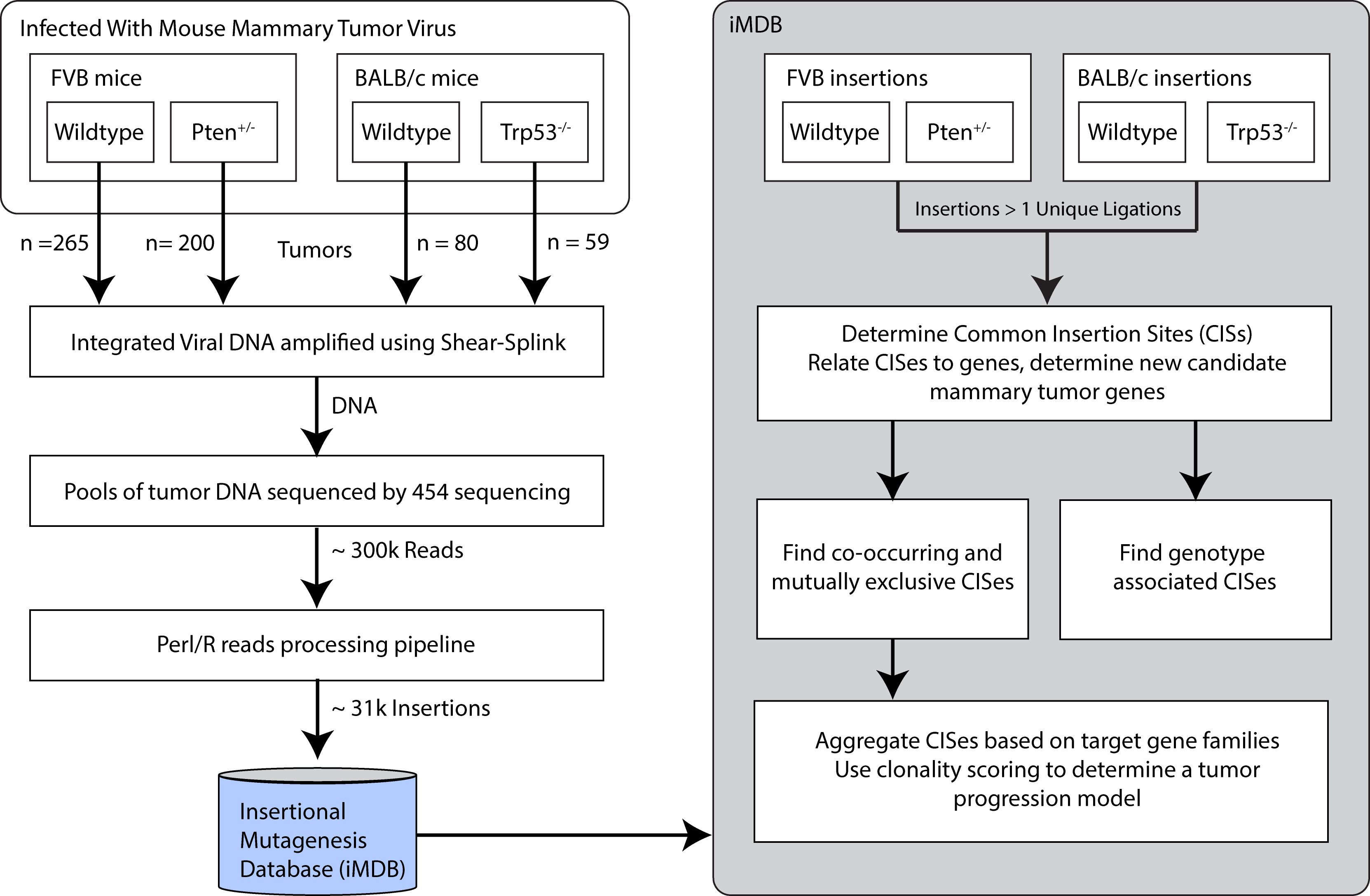

Supplement: Figure S1 — Schematic overview of the study. Boxes depict application of protocols and arrows indicate the flow of the resulting products. The gray box indicates that all processes take place inside the insertional Mutagenesis Database. (TIF) [file pone.0062113.s001.tif]

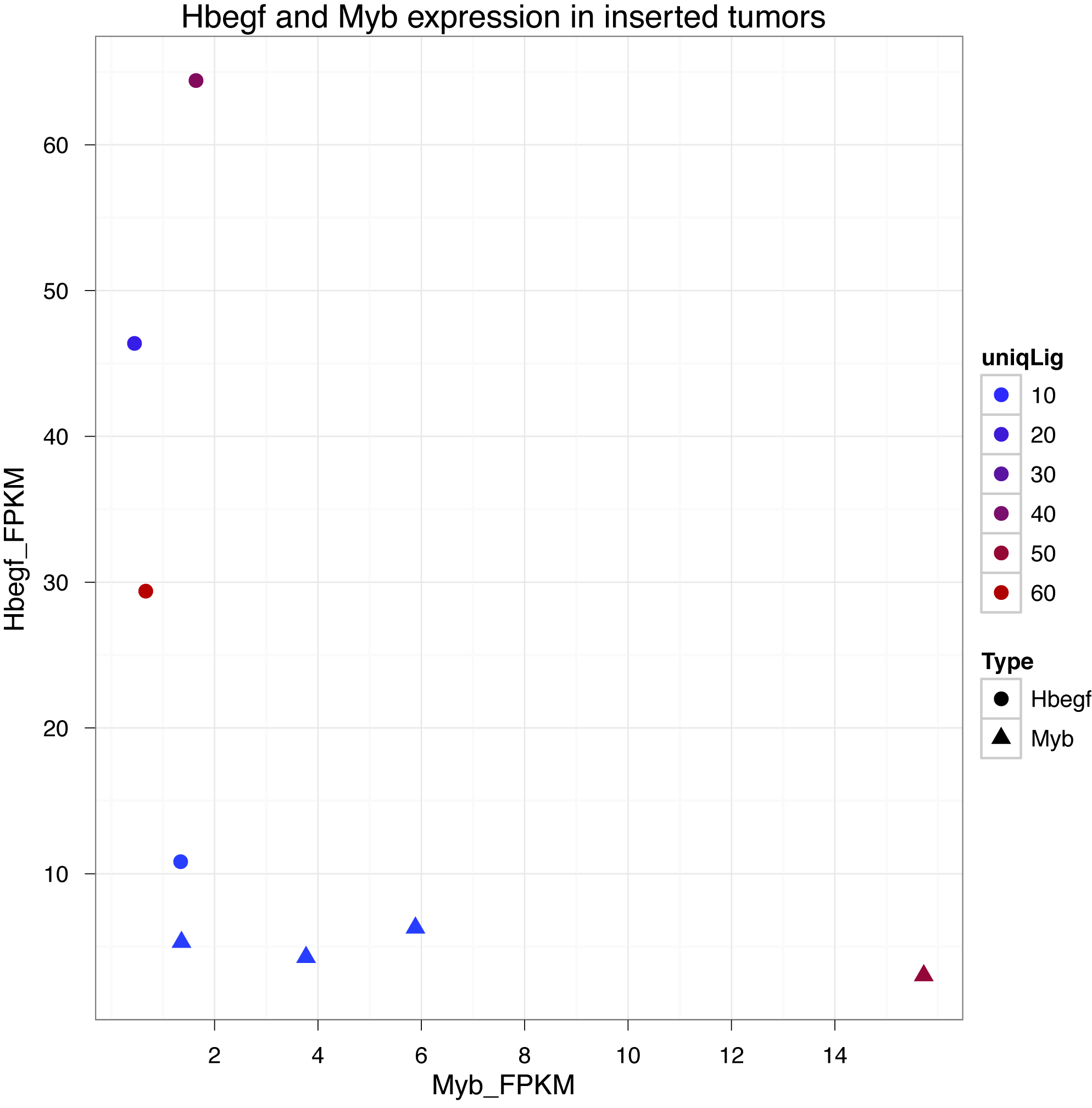

Supplement: Figure S2 — Expression of Myb and Hbegf in tumors with viral insertions near to these genes. The RPKM gene expression values of the genes Myb and Hbegf have been plotted for 8 mammary tumors. Four of these tumors contained a MMTV insertion near Myb (shown with triangles) and four of the tumors contained a MMTV insertion near Hbegf (shown with circles). The color of the symbols represents the clonality of those insertions. (TIF) [file pone.0062113.s002.tif]
