# Supplementary material for: Analysis of Tumor Heterogeneity and Cancer Gene Networks Using Deep Sequencing of MMTV-Induced Mouse Mammary Tumors
Source: PLoS One. 2013 May 14;8(5):e62113. doi: 10.1371/journal.pone.0062113 (PMC3653918; doi:10.1371/journal.pone.0062113)
Supplement: Text S1 — Supplemental Methods. This document describes the data analysis steps used in this study in more detail. (DOC) [file pone.0062113.s004.doc]

# Supplemental Methods - Analysis of tumor heterogeneity and cancer gene networks using deep sequencing of MMTV-induced mouse mammary tumors

Christiaan Klijn^1,3,#,†^, Marco J. Koudijs^1,#, ††^ , Jaap Kool^2,#, †††^, Jelle Ten Hoeve^3,#^, Mandy Boer^2^, Joost de Moes^2^, Waseem Akhtar^2^, Martine van Miltenburg^1^, Annabel Vendel-Zwaagstra^2^, Marcel J.T. Reinders^4^, David J. Adams^5,*^, Maarten van Lohuizen^2,*^, John Hilkens^2,*^, Lodewyk F.A. Wessels^3,4,*^, Jos Jonkers^1,*^.

^1^Division of Molecular Pathology, ^2^Division of Molecular Genetics, ^3^Division of Molecular Carcinogenesis, The Netherlands Cancer Institute, Plesmanlaan 121 1066 CX, Amsterdam, The Netherlands
^4^Delft Bioinformatics Lab, Delft University of Technology, Mekelweg 4, 2628 GA, Delft, The Netherlands
^5^Wellcome Trust Sanger Institute, Hinxton, Cambridge CB10 1SA, UK
^#^These authors contributed equally to this work
^†^Current address: Genentech, Department of Bioinformatics and Computational Biology. 1 DNA Way, 94080 South San Francisco, California, USA.
^††^Current address: UMC Utrecht, Department of Medical Oncology, Heidelberglaan 100, 3508 GA, Utrecht, The Netherlands
^†††^Current address: Intervet International bv, Wim de Körverweg 35, 5830 AA, Boxmeer, The Netherlands
^*^Correspondence: D.J.A. ([da1@sanger.ac.uk](mailto:da1@sanger.ac.uk)), M.L. ([m.v.lohuizen@nki.nl](mailto:m.v.lohuizen@nki.nl)) , J.H. ([j.hilkens@nki.nl](mailto:j.hilkens@nki.nl)) , L.F.A.W. ([l.wessels@nki.nl](mailto:l.wessels@nki.nl)), J.J. ([j.jonkers@nki.nl](mailto:j.jonkers@nki.nl))

##

## Data Pre-processing

All data preprocessing was performed using Perl and R scripts. Raw reads obtained from 454 sequencing were first examined for the presence of the MMTV 5’ LTR sequence, barcode (index for individual tumors) and T7 Splinkerette sequence using Exonerate (1). Subsequently, the reads were mapped to the mouse reference genome (NCBI m37 assembly) using BLAT (95% similarity). A new reference sequence was constructed from the correctly mapped reads, and a second round of mapping was performed to find add additional reads to mapped integration sites. We aggregated reads into insertion sites by clustering them on genomic coordinates using a 10 bp cutoff. Insertions sites containing reads from multiple tumors were assigned to a single tumor if more than 80% of the reads originated from that single tumor, otherwise they were discarded. Per insertion we counted the unique genomic coordinates of ligation to the Splinkerette sequence. This score is the clonality score. If no reads in an insertion contained the Splinkerette sequence we set the clonality score to 1. The data preprocessing is not part of the iMDB.

### Insertional Mutagenesis Database (iMDB)

In order to fully exploit the increased efficiency of IM screens by using shear-splink we use a new analysis platform with the capability to handle high-content IM datasets while also offering analysis tools to users to address the most common research questions related to these data. It is designed to handle clonality scores for insertions, based on the number of unique LP’s. For finding statistically significant CISs, we implemented the Gaussian Kernel Convolution (GKC) approach (2). Furthermore, we developed tools for calling significantly co-occurring or mutually exclusive insertions, implemented a tool to associate insertions with potential target genes and developed a module to combine insertional mutagenesis data with gene expression data of the same samples (3). Users are allowed to upload their own data, which is kept private, and perform these analyses on the data. All data presented in this paper are publically accessible from the iMDB. The iMBD can be accessed from imdb.nki.nl (4).

## Data Analysis

All data analysis was performed using the insertional mutagenesis database (iMBD) unless otherwise specified. To filter passenger insertions we restricted the analysis to insertion sites with two or more unique ligation points. Common insertion sites (CISs) were determined by the Gaussian Kernel Convolution method (2). We used a kernel width of 30 kbp and we calculated the significance at p < 0.05 (Bonferroni corrected over the number of peaks). For each insertion site a target gene was defined by manual curation. CISs were manually aggregated in gene groups based on the family of the curated target gene. We manually removed five CISs that mapped to endogenous MMTV fragments. If a gene had no family members among the CIS target genes, it would be the sole constituent of a gene group. Association of a CIS with a genotype was tested using a Fisher’s exact test. P values were corrected using the Benjamini-Hochberg FDR approximation. An association of p < 0.05 was called a significant genotype association.

We calculated co-occurrence and mutual exclusiveness of CISs: for each CIS pair (CIS-A and CIS-B) we counted the number of tumors containing an insertion in (1) CIS-A and CIS-B, (2) only in CIS-A, (3) only in CIS-B, (4) neither CIS-A nor CIS-B. By applying a left-sided and a right-sided Fisher’s exact test, we tested for co-occurrence and mutual exclusiveness, respectively.

We used the aggregated gene groups with in total more than ten inserted tumors to test for a clonality relation. To consider the clonality relation between two gene groups we regarded only those tumors with insertions in both gene groups. If, for a single gene group, multiple CISs had integrations we only regarded the CIS with the highest clonality score. For each tumor with an insertion in both gene groups we scored whose clonality score was higher. Using a binominal test (as implemented in R) with p = 0.5 we calculated whether one of the two gene groups had a higher clonality score more frequently than could be expected by chance. We constructed a directional network of gene groups with a significant difference in clonality scores with P < 0.05.

## References

1. Slater G, Birney E. Automated generation of heuristics for biological sequence comparison. BMC Bioinformatics. 2005;6(1):31.

2. de Ridder J, Uren A, Kool J, Reinders M, Wessels L. Detecting statistically significant common insertion sites in retroviral insertional mutagenesis screens. PLoS Comput. Biol. 2006 Dec 8;2(12):e166.

3. de Ridder J, Gerrits A, Bot J, de Haan G, Reinders M, Wessels L. Inferring combinatorial association logic networks in multimodal genome-wide screens. Bioinformatics. 2010 Jun 15;26(12):i149–157.

4. Koudijs MJ, Klijn C, Van Der Weyden L, Kool J, Ten Hoeve J, Sie D, et al. High-Throughput Semiquantitative Analysis of Insertional Mutations in Heterogeneous Tumors. Genome Res. 2011 Dec 1;21(12):2181–9.
